# Testicular adrenal rest tumor in a pediatric patient with congenital adrenal hyperplasia: A case report

**DOI:** 10.1016/j.radcr.2023.08.081

**Published:** 2023-09-14

**Authors:** Mohammad G. Ibdah, Salem M. Tos, Narmeen Giacaman, Anas Aljundi, Mohamad Qabaja, Muayad Salman

**Affiliations:** a Al-Quds University, College of Medicine, Palestine; b Radiology Department, Al-Makassed Hosptial, Jerusalem, Palestine

**Keywords:** Testicular adrenal rest tumor, TART, Testicular tumor, Congenital adrenal hyperplasia

## Abstract

Testicular adrenal rest tumors (TARTs) are benign intratesticular tumors that occur mostly in male patients with congenital adrenal hyperplasia (CAH), their prevalence in these populations can reach up to 94%. We hereby report a male child with known CAH, presented with bilateral irregular testicular masses which were diagnosed as TARTs. TARTs were first reported in 1940, They were named due to their resemblance to adrenal tissue, they are almost always benign but can blunt spermatogenesis and endocrine function of the testis leading to infertility, they are diagnosed by a combination of clinical history, physical exam, and imaging studies, MRI and U/S are equally good for diagnosis and follow-up, treatment includes surgical resection or observation depending on tumor size, symptoms, and fertility goals. TARTs are benign testicular tumors that are strongly associated with CAH, they can be completely asymptomatic or can cause pain and infertility, diagnosis can be done by imaging modalities like MRI or U/S, and treatment options include observation or surgical removal.

## Introduction

Testicular adrenal rest tumors (TARTs) are benign intratesticular growths that develop in male patients with congenital adrenal hyperplasia (CAH), a genetic disorder characterized by impaired cortisol synthesis, most commonly caused by a deficiency in 21-Hydroxylase. TARTs have an average prevalence of 40% in male CAH patients [1,2] some reports described prevalence as high as 94% [3].

TARTs are hypothesized to originate from a pluripotent steroidogenic cell type that is present in utero, probably from adrenogonadal primordium or urogenital ridge, they histologically resemble adrenal cells, hence the name. TARTs are benign but can blunt both spermatogenesis and endocrine function of the testis, and are considered the most common cause of infertility in male patients with CAH [2].

## Case presentation

A 6-year-old male known to have CAH due to 11-hydroxylase deficiency, with unremarkable antenatal and perinatal history, a product of normal vaginal delivery, weight of 3400 grams. Lately, his parents noted that he has a large penis, and had developed axillary hair, after laboratory and genetic testing he was diagnosed with CAH.

**Fig. 1:**
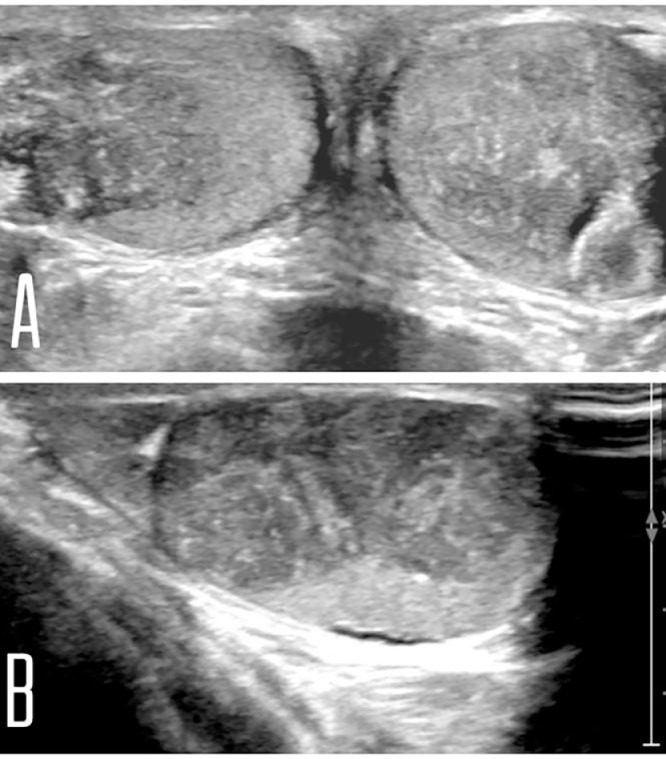
Scrotal ultrasound (A, both testicles, B, right testicle): shows average size testicles with a focal, irregular, eccentric, heterogeneous hypoechoic lesion adjacent to the mediastinum testis in both testicles. No mass effect, narrowing, or distortion is seen traversing through these lesions.

**Fig. 2:**
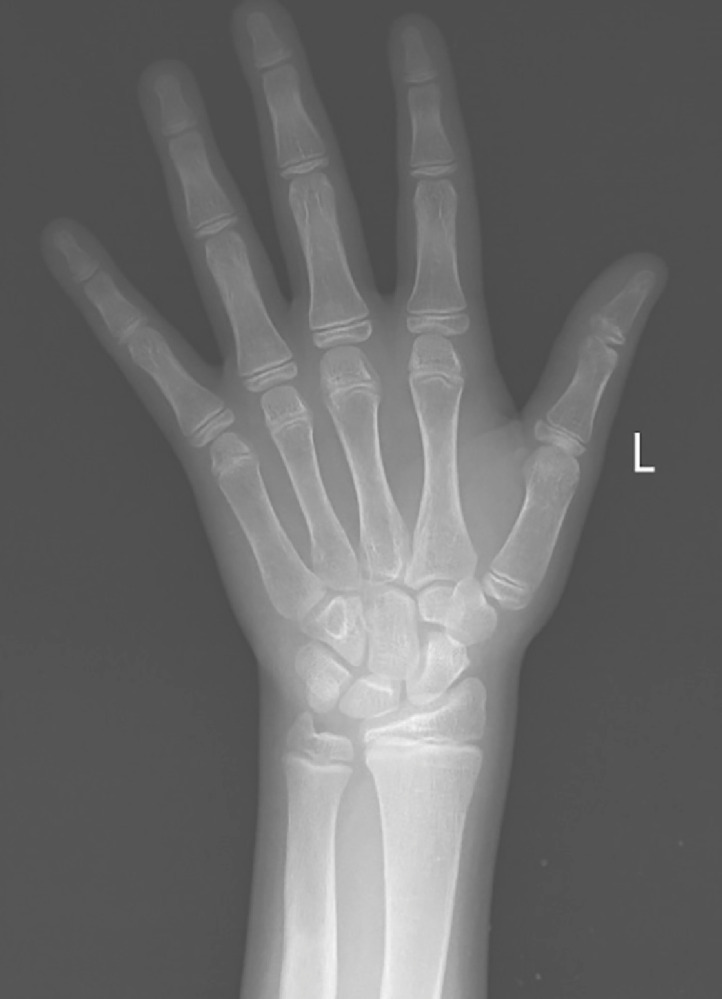
X-ray of the left hand shows the bone age matches the reference standard of 13 years with a standard deviation of +/-12 months (according to Greulich and Pyle atlas). At that time, the patient's chronological age was 6 years (indicating significant advanced bone age).

Physical examination revealed a large penis, gynecomastia, and bilateral irregular testicular masses were palpated. Laboratory tests were as follows: low LH, low FSH, elevated 17-hydroxyprogesterone, blood count, renal and liver function tests, and tumor markers (alpha-fetoprotein, beta-hCG) all were within normal limits. Scrotal U/S showed the following (Fig. 1) and bone age was done, which showed the age of 13 years at the chronological age of 6 years (Fig. 2).

Diagnosis of TART was made principally by its radiological features, as U/S showed [1] Bilateral testicular lesions [2], Located near the mediastinum [4], Clear boundary [5], which are typical for TART, in addition to the patient history of CAH with further supported the diagnosis.

## Discussion

TARTs are benign intratesticular masses that are most frequently detected in male patients with CAH, which is majorly caused by a deficiency in 21-hydroxylase in more than 90% of cases, TARTs have an average prevalence of 40% in males of CAH [1,2].

TARTs were first reported in 1940 by Wilkins et al [6], they were named this because of their morphological resemblance to adrenal tissue, they were always benign, but commonly can lead to azoospermia and infertility, due to tumor compression and obstruction of seminiferous tubules which can lead to irreversible damage [7].

TARTs usually occur during childhood or adolescence, and their prevalence is higher in individuals with the more severe forms of CAH, the youngest age at which a TART was detected was 1.8 years [4] The exact cause of them is not fully understood, but it is believed to result from abnormal migration of adrenal tissue during fetal development, it is hypothesized that TARTs originate from a pluripotent steroidogenic cell type that is present in utero, probably from adrenogonadal primordium or urogenital ridge, they histologically resemble adrenal cells, hence the name [2].

TARTs are benign but can blunt both spermatogenesis and endocrine function of the testis, and considered the most common cause of infertility in male patients with CAH, this effect is proportionate to the tumor size [4].

Macroscopically, well-established TARTs are rigid and multilobular with a yellow color on the cut surface, they are most commonly located within the rete testis, and in most patients, they are present bilaterally [8].

An important differential diagnosis in cases of TARTs is the malignant Leydig cell tumors, as histological differentiation can be challenging. Still, some clinical features can help to differentiate between these two. Fundamentally, TARTs as we said earlier tend to be bilateral, up to 80% of the time, on the other hand, Leydig cell tumors are bilateral only in 3% of cases. Also, the presence of Reinke crystals supports the diagnosis of Leydig cell tumors which can be found in up to 40% of them. Malignant degeneration is seen in Leydig cell tumors but never in TARTs. Finally, the classic location of TARTs at rete testis can also further help to make the differentiation [9,10].

Symptoms of TARTs can vary and may include testicular pain, swelling, or a palpable lump. However, some individuals may not experience symptoms, and the tumors may be discovered incidentally during routine medical examinations or imaging tests.

**Fig. 3:**
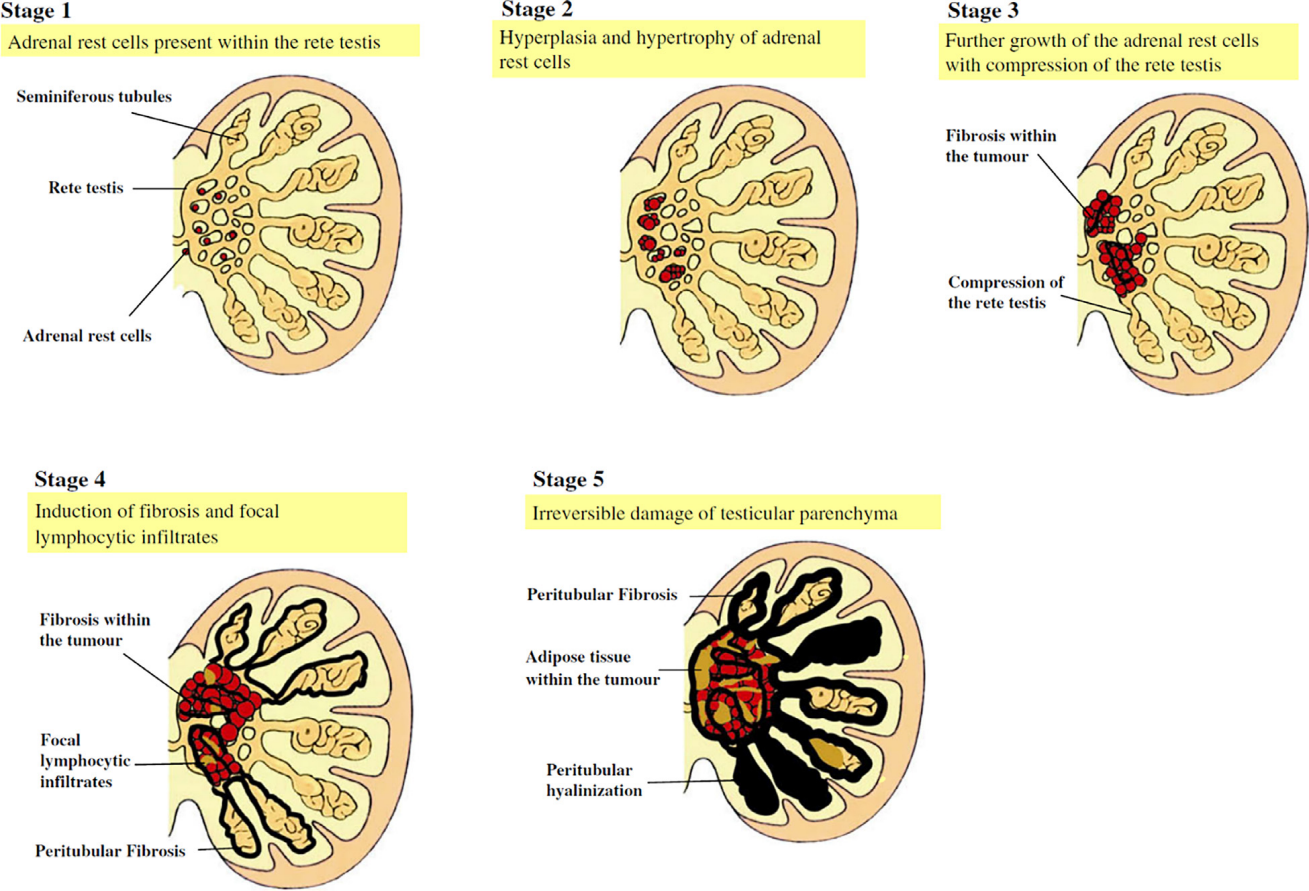
Picture copyright: H.L. Claahsen-van der Grinten et al. / Best Practice & Research Clinical Endocrinology & Metabolism 23 (2009) 209–220.

TARTs are classified into 5 different stages, based on their histological appearance and involvement of testicular parenchyma [7], they are described briefly in Fig. 3, noteworthy, stage 1 cannot be detected even with radiological approaches.

Diagnosis of TARTs typically involves a combination of medical history evaluation, physical examination, although most tumors are nonpalpable and only can be palpated when they reach > 2 cm [10]. Gonadal function evaluation can also help to determine the degree of testicular failure, it includes testing blood LH, FSH, inhibin B and testosterone concentration [11].

Imaging modalities like MRI and U/S are equally good for detection and follow-up, but the latter is preferable since it is cheap and quick, and even very small TARTs of a few millimeters can be visible [8]. Diagnosis of TART can be confirmed by biopsy with the utilization of various immunohistochemistry stains like CD56, inhibin, and vimentin which are typically positive [12].

On U/S examination of TARTs, they are classically irregular, lobulated, hypoechogenic masses, located adjacent to the testicular mediastinum, which is similar to our case finding (Fig. 1).

Rarely these masses can be hyperechogenic, indicating chronicity, fibrosis, and calcifications. Doppler studies typically illustrate rich blood supply [12], in addition, vessels crossing through the lesion do not deviate or change in caliber, this observation which was made by Avila et al [5] can be useful in differentiating between TARTs and other testicular tumors as they lack this feature.

Treatment options for TARTs depend on several factors, including the size and location of the tumors and the individual's symptoms and fertility goals. TARTs have no malignant features, and it seems that there is no need to excise them at early stages. In some cases, observation and regular monitoring may be sufficient if the tumors are small and not causing significant symptoms. However, surgical removal may be necessary if the tumors are large, causing pain or affecting fertility [13].

## Conclusion

Testicular adrenal rest tumors are benign growths that develop in the testicles of individuals with congenital adrenal hyperplasia. They are typically associated with a hormonal imbalance caused by CAH and can cause symptoms such as testicular pain or swelling. Diagnosis involves medical evaluation and imaging studies, while treatment options include observation or surgical removal.

## Ethical approval

Informed consent was signed by the patient's parents for publication.

## Provenance and peer review

Not commissioned, externally peer-reviewed.

## Authors’ contributions

Study concept or design: Muayad Salman. Writing the manuscript: Mohammad G. Ibdah, Salem M.Tos, Narmeen Giacaman, Anas Aljundi, Mohamad Qabaja. Review & editing the manuscript: Mohammad G. Ibdah, Muayad Salman.

## Patient consent

A written informed consent was obtained from the patient's parents for publication of this case report (*Testicular adrenal rest tumor in a pediatric patient with congenital adrenal hyperplasia: a case report*) and accompanying images. A copy of the written consent is available for review by the Editor-in-Chief of this journal on request.
